# Better Understanding the Workers' Retirement Decision Attitudes: Development and Validation of a New Measure

**DOI:** 10.3389/fpsyg.2018.02429

**Published:** 2018-12-04

**Authors:** Evelyne Fouquereau, Grégoire Bosselut, Séverine Chevalier, Hélène Coillot, Virginie Demulier, Caroline Becker, Nicolas Gillet

**Affiliations:** ^1^EA 2114 Psychologie des Âges de la Vie, University of Tours, Tours, France; ^2^Laboratoire Epsylon EA 4556, University of Montpellier, Montpellier, France; ^3^LIMSI-CNRS, University Paris-Sud, Orsay, France

**Keywords:** workers' retirement motivations inventory, WRMI, older workers, scale development, push pull anti-push anti-pull model

## Abstract

The aim of the present research was to develop a measure that could be used in future research for in-depth study of the psychological management of retirement. We report the results of six studies involving 1,898 French workers designed to develop and assess the psychometric properties of a new instrument named the Workers' Retirement Motivations Inventory (WRMI) using the push pull anti-push anti-pull model. The items were constructed based on a review of the relevant psychological literature and face-to-face interviews with senior workers. A combined method of exploratory structural equations modeling and confirmatory factor analysis (CFA) was employed and provided evidence for validating this structure of the inventory. The WRMI showed consistency of the four-factor structure across different samples, internal consistency, test-retest reliability, and predictive validity of workers' plans for retirement. Implications of these findings and avenues for counseling activities and future research are discussed.

## Introduction

In Europe, the number of retired people has increased significantly in recent decades and will continue to rise as the baby boom generation reaches retirement age (De Preter et al., 2013). At the same time, Western countries are experiencing a serious economic crisis. This clearly leads to a problematic disparity between an aging population and the state's economic needs (Dervis, 2013). Consequently, new measures have been introduced to enable older people to continue participating in the workforce, notably by raising the retirement age (typically between 60 and 65). In addition, organizations have developed incentives (e.g., training, flexible working hours; Dal Bianco et al., 2015) to encourage seniors to continue working (Van Solinge and Henkens, 2014). The success of these strategies is based on the assumption that older workers wish to continue working and to delay the end of their working life. However, surprisingly, little is known about the complex motivations of older workers to continue or to stop working. A better understanding of these motivations is thus needed in order to identify the key issues underlying retirement decisions (Kooij et al., 2008). This article aims to improve our understanding of the psychosocial conditions underlying workers' retirement decisions by developing a new instrument to explore this process in depth, based on the push pull anti-push anti-pull model (Mullet et al., 2000).

Wang and Shi (2014) recently defined retirement as an individual's exit from the workforce, which accompanies decreased psychological commitment to and behavioral withdrawal from work. This definition emphasizes that retirement does not just occur at a single point in time but is a psychological and behavioral process often evolving over a long period. More precisely, the temporal process model of retirement postulates that the retirement process involves three sequential phases: retirement planning, retirement decision-making and retirement transition, and adjustment (Shultz and Wang, 2011). Research shows that the first two phases (i.e., retirement planning and retirement decision-making) are crucial for predicting retirement adjustment (e.g., Earl and Archibald, 2014).

In psychological research, retirement has often been conceptualized as an informed decision-making process (e.g., Shultz and Wang, 2007; Wang and Shultz, 2010). According to some scholars, when workers decide to retire, they make a motivated choice based on the information they have about their own characteristics and their work and non-work environment (Wang and Shi, 2014). These factors underlying workers' decisions to retire and their wide variety of consequences (e.g., Pinquart and Schindler, 2007; Wang and Shultz, 2010; Leung and Earl, 2012) have been a focus of concentrated investigation in recent years (Johnson, 2009). For example, the strong connection between poor self-rated health, the decision to retire early, and difficulty adjusting to post-professional life has been widely demonstrated (Damman et al., 2011). Based on this approach to retirement as an informed decision-making process, a number of theories (e.g., rational choice theory, expectancy theory) have further identified the characteristics of the decision process. For example, in rational choice theory, the retirement decision is viewed as the result of comparing the financial resources that have been accumulated with those needed in retirement (Laitner and Sonnega, 2013). More generally, according to this approach, individuals retire when the benefits are maximized and the costs minimized (Hechter and Kanazawa, 1997). By contrast, in expectancy theory, job characteristics and subjective life expectancy can affect the decision to retire (e.g., van Solinge and Henkens, 2010). These theoretical models have provided comprehensive frameworks for understanding the retirement decision process.

However, on the one hand, the increasing uncertainty of the context surrounding retirement (Feldman and Beehr, 2011) has made the decision to retire more challenging. Secondly, these theoretical models differ with regard to the weight attributed to each contributing factor and consequently underline the difficulty of identifying the complexity of the individual retirement decision process. To clarify the multi-factorial nature of this process, the literature distinguishes between the push and pull factors influencing older workers' decisions to retire (Feldman, 1994; Adams and Beehr, 2003; Noone et al., 2013). Push factors have been defined as negative considerations that induce older workers to retire, such as excessive workload (Kim and Feldman, 1998). By contrast, pull factors are typically positive considerations that motivate older workers to retire, such as the desire to pursue voluntary activities (Shultz et al., 1998; Van Oorschot and Jensen, 2009). In brief, consensus has been reached that the act of retirement is based upon different types of reason linked to both current and future life, which interact in complex ways to influence a person's anticipation and intention to retire (e.g., Shultz et al., 1998; Van Oorschot and Jensen, 2009). But while the studies based on the *Push Pull* framework have investigated the factors that lead workers to retire (i.e., levers to the retirement decision), they have not examined why people choose to continue working (i.e., barriers to the retirement decision).

Mullet et al. (2000) were the first authors to examine both present vs. future levers and present vs. future barriers in the decision-making process of young French people deciding whether or not to study or work abroad. To that end, they added two other dimensions to those cited above, namely anti-push and anti-pull factors. The anti-push factor has been conceptualized as attachment to the present situation, and the anti-pull factor as the perceived costs and risks of the future situation. In summary, the authors emphasized that this model was particularly relevant for understanding a complex decision process, whatever the context.

More recently, two self-report instruments based on this theoretical push pull anti-push anti-pull model have been developed in the specific area of career termination, in order to explain (1) the retirement decision process of competitive athletes (Fernandez et al., 2006) and (2) the retirement decision process of entrepreneurs (Chevalier et al., 2013). The findings of these new reliable instruments emphasize the suitability of the push pull anti-push anti-pull model to improve understanding of the retirement process by examining the positive and negative aspects of both the present and the future situation (Chevalier et al., 2017). However, the pattern of reasons for retirement is obviously linked to the characteristics of the populations studied. Consequently, precise assessment of the structure of the reasons that lead workers to retire from employment requires a relevant, specific tool based on the push pull anti-push anti-pull framework. This type of multidimensional tool could help account for the complex process of deciding to retire by showing how an employee may for example be pulled to retirement by the desire to discover new opportunities, and at the same time be afraid of feeling useless without employment (i.e., anti-pull factor). Conversely, workers may be pushed toward retirement because of company restructuring, and at the same time feel convinced that they could still be useful for the company (i.e., anti-push factor).

Thus, the primary aim of this study was to develop a measure that could be used in future research for in-depth study of the psychological management of career termination. To date, studies about the determinants of the retirement decision process have mainly focused on a subset of dimensions identified by the researchers, without taking into account the findings of empirical studies that the retirement decision is the result of an interaction and balance between push, pull, anti-push, and anti-pull factors. This new tool could also be very useful for career counselors who help workers prepare for retirement, enabling them to diagnose the importance of each factor in the retirement decision and consequently to set up more targeted counseling strategies.

## The Present Research

The aim of the present research was to develop and validate a new instrument to assess the subjective reasons underlying workers' retirement decisions. This inventory, based on the push pull anti-push anti-pull model, was named the Workers' Retirement Motivations Inventory (WRMI). The development and validation procedure included six studies involving a total of 1,898 French workers close to retirement (aged over 50) from various sectors (e.g., business, industry or service). The first study (i.e., pilot study) aimed to create a pool of items intended to constitute the frame of the WRMI for subsequent testing. The second study involved a test of the factor structure and the construct validity of the items. In the third study, the factorial structure of the 20-item inventory was tested with a new sample using confirmatory factor analysis (CFA). The fourth study evaluated the impact of social desirability on responses to the WRMI, and simultaneously investigated its test-retest reliability. The fifth study analyzed the measurement invariance of the inventory across several individual characteristics (i.e., gender, marital status, contract, age, and tenure). Finally, study 6 examined the predictive validity of the WRMI. Data were gathered from 2015 to 2017. Apart from the first qualitative study, participants completed the scales via an online inventory. The ethical conditions were the same for all the studies. According to local regulations, no formal ethical scrutiny was required, as no ethics committee existed in the institution at the time of these studies. However, participation was voluntary and participants gave their informed consent. They were told that their answers would remain anonymous and confidential and that they could withdraw from the study at any time.

We describe below in detail the studies that we conducted to validate the WRMI.

## Validation Studies

### Study 1: Item Screening and Development

The purpose of Study 1 was to develop a preliminary pool of items. In accordance with Hinkin's (1998) guidelines for validation studies, a broad review of the relevant psychological literature was first conducted in order to cover the field of the specified domain (i.e., reasons underlying workers' retirement decisions). Secondly, to heighten our understanding of the individual process we conducted face-to-face semi-structured interviews.

#### Method

Data were collected from 40 French workers (24 men and 16 women), aged 55–62 years (*M*_*age*_ = 59.1 years; *SD*_*age*_ = 1.72). They worked in various sectors (e.g., business, industry or service). To ensure that the final version of the WRMI would be sensitive to the experiences of people undergoing the transition from working life to retirement, we only recruited individuals who estimated they were close to retirement (within 5 years).

The interviewers were four psychology graduates trained in interviewing techniques. Questions were based on the Push Pull Anti-push Anti-pull model. The participants were asked the four following open questions: (a) “What reasons would lead you to end your professional career?,” (b) “What aspects of life would make you want to retire?,” (c) “What reasons would encourage you to continue working?,” and (d) “What particular worries do you have about retiring?”

The interviews lasted approximately 1 h during which the participants were encouraged to speak freely about their transition from vocational activity. The interviews were tape-recorded and transcribed to ensure accuracy and to provide extensive data. Content analysis techniques were applied to classify the textual information into a smaller number of relevant content units (Krippendorff, 2004). Categorization was based on a phenomenographic approach, which investigates the qualitatively different ways of perceiving a phenomenon (Fülop, 2004). A panel of three researchers specialized in career termination used previous literature in this research area based on the Push Pull framework (e.g., Shultz et al., 1998; Brougham and Walsh, 2007; Potocnik et al., 2009) to identify and categorize raw data into significant themes and patterns that could represent possible reasons for staying in or terminating the vocational career.

#### Analysis and Results

A set of 88 potential items was generated from the literature and the interviews. More precisely, 74 percent of items were derived from the literature (e.g., “Being afraid of being bored when I retire,” Maggiori et al., 2014) and 36 percent were obtained from interviews using content analysis (e.g., “Feeling that I can still play an active role at work”). We followed guidelines for item wording to make them as precise, clear, and short as possible (Clark and Watson, 1995). Next, this set of items was pilot tested with 12 additional participants (8 men and 4 women) aged 55–62 years (*M*_*age*_ = 58.7 years; *SD*_*age*_ = 2.21) who rated the clarity of each item on a 5-point Likert scale ranging from 1 *(“totally unclear”)* to 5 *(“totally clear”)*. Using the ratings and the comments provided by these participants, two items were slightly rewritten. In this way, 88 items were retained and constituted an initial version of the WRMI.

### Study 2: Initial Content Validity

The purpose of Study 2 was to explore the factorial composition and structure of the new scale. Item reliability was also investigated. To enable the WRMI to be used in combination with other instruments in future research, we decided to develop a short scale with 5 items in each factorial dimension (i.e., push, pull, anti-push, anti-pull dimensions) to ensure that the inventory had adequate internal consistency and validity while maintaining reasonable length (Tabachnick and Fidell, 2013).

#### Method

##### Participants

A total of 251 workers (128 men and 123 women) from various sectors of activity in France (e.g., business, industry, or service) took part in the study. Ages ranged from 45 to 62 years with an average of 52.40 years (*SD* = 3.33). Participants worked full-time (86%) or part-time (13%) (4 workers did not indicate their working hours), and their average tenure was 33.24 years (*SD* = 4.92).

##### Measure

Each participant completed the preliminary 88-item version of the WRMI using a 10-point Likert-type scale ranging from 1 (*not important at all*) to 10 (*very important*). Instructions given to participants were “When you decide whether to retire, how important would each of the following reasons be for you?” for the push and pull items, and “When you decide whether to continue working, how important would each of the following reasons be for you?” for the anti-push and anti-pull items.

#### Statistical Analysis and Results

In accordance with the recommendations of Tabachnick and Fidell (2013), univariate (i.e., |z| < 3.29, *p* < 0.001, two-tailed test) outliers were excluded from the analyses. In this way, 236 participants were included in the analyses described below. Some of the participants did not respond to all 88 items of the WRMI, but the percentage of missing values was lower than 5% and thus did not represent a problem (e.g., Graham and Hofer, 2000). When testing for missing values, Little's chi-square test for MCA was not significant (*p* > 0.05), Chi^2^ (244) = 295.692 indicating that data are missing completely at random. The expectation maximization (EM) algorithm was thus used to impute missing values using SPSS software (version 18).

First, we carried out an exploratory factor analysis with oblique rotation. Oblique rotation allows for correlated factors, as we might expect that at least, pull and push in the first hand and anti-pull and anti-push in the second hand would be correlated (Kim and Lee, 2002; Chevalier et al., 2013). To determine how many factors to retain, we used both the scree plot and the eigenvalues-greater-than-one rule (Cattell and Vogelmann, 1977). Examination of the scree plot showed clear discontinuity in the slope after four factors, suggesting that extracting four factors was appropriate (Tabachnick and Fidell, 2013). Moreover, only these four factors had eigenvalues >1, more precisely between 1.77 and 4.70. They explained 45.14% of the variance of the items. In order to bring the WRMI down to 5 items per factor to make it parsimonious while maintaining reliability, stability, and interpretability (Tabachnick and Fidell, 2013), we selected the five highest loading items on each factor, following a successful approach used in previous studies (e.g., Eisenberger et al., 2002). We checked that the content of the selected items was not redundant.

A second exploratory factor analysis was then carried out. The oblique rotation converged in 6 iterations with eigenvalues of 3.74, 3.26, 2.21, and 1.53 for the first, second, third, and fourth factors, respectively. The percentages of variance accounted for by the four factors were 18.71, 16.32, 11.09, and 7.66. The four factors explained 53.78% of the variance of the final 20-item version of the WRMI. Items had factor loadings ranging from 0.59 to 0.84, and no cross-factor loadings (i.e., >0.40 on only one factor). An examination of the interpretability of the factors showed that the first factor corresponded to the push dimension (e.g., “*Etre moins motivé(e) dans mon travail*” [being less motived in my job]), the second referred to the anti-pull dimension (e.g., “*Avoir peur de déprimer, une fois à la retraite*” [being afraid of being depressed when I retire]), the third expressed the pull dimension (e.g., “*Pouvoir passer plus de temps avec mes ami(e)s, une fois à la retraite*” [being able to spend more time with my friends when I retire]), and the fourth referred to the anti-push dimension (e.g., “*Avoir encore des ambitions professionnelles*”[still having professional ambitions]). Items are reported in Supplementary Material. Descriptive statistics of each item and each dimension are presented in Table 1, and inter-item correlations and inter-factor correlations in Table 2. Finally, acceptable Cronbach's alphas were obtained (Nunnally, 1978) with values between 0.72 and 0.83 (see Table 1).

**Table 1:** Means, standard deviations, and standardized factor loadings from the exploratory factor analysis (Study 2).

		***M***	**SD**	**α**	**Push**	**Pull**	**Anti-push**	**Anti-pull**
1	Push	5.95	2.17	0.83				
	1a	5.40	2.88		**0.82**	−0.07	0.01	0.06
	1b	5.89	2.62		**0.70**	−0.01	−0.01	0.01
	1c	6.69	2.85		**0.76**	0.13	−0.01	−0.05
	1d	5.58	2.84		**0.84**	−0.04	0.01	0.03
	1e	6.18	2.88		**0.68**	0.19	0.03	−0.01
2	Pull	7.73	1.47	0.73				
	2a	6.94	2.77		−0.05	**0.61**	−0.02	−0.08
	2b	8.40	1.79		0.02	**0.76**	−0.03	−0.10
	2c	8.31	1.85		0.03	**0.75**	−0.11	0.05
	2d	7.85	1.86		0.07	**0.65**	0.08	−0.02
	2e	7.18	2.57		0.13	**0.59**	0.07	0.12
3	Anti-push	5.51	1.93	0.72				
	3a	6.26	2.69		−0.05	0.16	**0.59**	0.08
	3b	6.07	2.85		0.07	−0.01	**0.71**	0.08
	3c	4.92	2.70		−0.19	0.14	**0.54**	0.17
	3d	4.62	2.99		−0.01	−0.13	**0.67**	−0.16
	3e	5.69	2.67		0.07	−0.06	**0.79**	−0.05
4	Anti-pull	4.34	2.04	0.79				
	4a	4.41	2.81		0.06	−0.03	−0.07	**0.82**
	4b	4.40	2.78		0.08	−0.07	−0.08	**0.85**
	4c	5.54	3.03		−0.17	0.11	0.09	**0.61**
	4d	3.78	2.54		0.06	−0.08	0.18	**0.56**
	4e	3.95	2.70		−0.05	−0.02	−0.02	**0.80**

*Loadings in bold are values > 0.40*.

**Table 2:** Inter-item correlations and inter-factor correlations (Study 2).

		**1b**	**1c**	**1d**	**1e**	**2**	**2b**	**2c**	**2d**	**2e**	**3**	**3b**	**3c**	**3d**	**3e**	**4**	**4b**	**4c**	**4d**	**4e**
1	Push					0.29^**^					0.04					−0.21^**^				
	1a	0.46^**^	0.52^**^	0.54^**^	0.47^**^															
	1b	_	0.48^**^	0.50^**^	0.37^**^															
	1c		_	0.58^**^	0.51^**^															
	1d			_	0.49^**^															
2	Pull					_					−0.02					0.01				
	2a						0.35^**^	0.30^**^	0.21^**^	0.18^**^										
	2b						_	0.43^**^	0.42^**^	0.33^**^										
	2c							_	0.34^**^	0.38^**^										
	2d								_	0.35^**^										
3	Anti-pull										_					0.25^**^				
	3a											0.67^**^	0.32^**^	0.41^**^	0.51^**^					
	3b											_	0.34^**^	0.36^**^	0.60^**^					
	3c												_	0.32^**^	0.39^**^					
	3d													_	0.37^**^					
4	Anti-push															_				
	4a																0.38^**^	0.29^**^	0.20^**^	0.42^**^
	4b																_	0.43^**^	0.31^**^	0.41^**^
	4c																	_	0.28^**^	0.35^**^
	4d																		_	0.45^**^

** *p < 0.01*.

Thus, the results of Study 2 provide initial support for the reliability and factorial validity of the 20-item version of the WRMI.

### Study 3: Confirmation of Factor Structure Validity

The purpose of Study 3 was to administer the WRMI to a separate sample to test the factorial structure of the items selected in Study 2 via CFA. As mentioned above, a four-factor model was hypothesized, but following the suggestions of Mulaik et al. (1989), who stressed that well-fitting models may suffer from misspecification, alternative models based on past literature were also tested.

#### Method

##### Participants

Data were collected from 375 workers (180 men and 195 women) from various French sectors of activity. Their average age was 52.63 years (*SD* = 3.27), ranging from 49 to 61 years, and average tenure was 32.62 years (*SD* = 5.85); 88.80% worked full-time and 11.20% part-time.

##### Measure

In line with Study 2, participants completed the 20-item inventory (i.e., WRMI). A 10-point Likert-type scale was used, anchored at the extremes by “not important at all” (1) and “very important” (10). Acceptable Cronbach's alphas were obtained for the four WRMI dimensions (Nunnally, 1978) with values between 0.70 and 0.80 (see Table 3). However, as mentioned by Raykov (1997), coefficient alpha always underestimates reliability when measurement errors are uncorrelated, and always overestimates it when measurement errors are correlated. To address this problem, the author suggested providing an alternative to alpha, namely the composite reliability coefficient (CRC). This estimates the extent to which a set of latent variable indicators are common to a construct. CRC above 0.70 is considered as an indicator of good reliability (Hair et al., 1998). Results indicated an acceptable reliability for each dimension of the WRMI, ρ = 0.78, ρ = 0.70, ρ = 0.76, and ρ = 0.85 for push, pull, anti-push, and anti-pull, respectively.

**Table 3:** Descriptive statistics for study 3.

		**Min**	**Max**	***M***	**SD**	**Skewness**	**Kurtosis**	**α**	**2**.	**3**.	**4**.
1	Push	1	10	5.66	2.05	0.37	−2.71	0.77	0.20^**^	−0.12^*^	0.11^*^
2	Pull	1	10	7.66	1.39	0.81	1.07	0.70	_	−0.01	−0.15^*^
3	Anti-push	1	10	5.52	1.96	−1.51	−1.01	0.76		_	0.26^**^
4	Anti-pull	1	10	4.55	2.04	−2.77	−2.38	0.80			_

* *p < 0.05*;

** *p < 0.01*.

#### Statistical Analysis and Results

Descriptive statistics are presented in Table 3. Fourteen univariate (i.e., |*z*| < 3.29, *p*<*0.0*01, two-tailed test) and two multivariate outliers (Mahalanobis distance lower than $\chi_{\left(4\right)}^{2}$ = 18.47, *p* < 0.001) were excluded, leaving 359 participants for the analyses. The sample had < 5% of missing values, and analysis indicated that these were completely random (i.e., Little's χ^2^ = 174.26, df = 613, *p* = 1.00). Therefore, the EM algorithm was used to impute missing values using SPSS software.

Since the variables were abnormal (z values for kurtosis ranging from −2.71 to 1.07; *z*-values for skewness ranging from −2.77 to 0.81; and Mardia's normalized skewness coefficient = 50.22 with a criterion ratio of kurtosis multinormality above 1.96), confirmatory factor analyses were performed using Bootstrapped Maximum Likelihood estimation with AMOS 20.0 software (Arbuckle, 2011). Thus, all fit values provided in this study were based on AMOS 20.0 Bollen-Stine bootstrap *p-*value and bootstrap adjusted chi-square and goodness-of-fit indexes (Fouladi, 2000; Yuan and Hayashi, 2003). Assessment of fit was based on multiple indicators: the Bollen-Stine χ^2^, the B-Sχ^2^/df ratio, the comparative fit index (CFI; Bentler, 1990), the Tucker-Lewis Index (TLI; Tucker and Lewis, 1973), the standardized root mean square residual (SRMR; Jöreskog and Sörbom, 2001), the root mean square error of approximation (RMSEA; Browne and Cudeck, 1989) and the 90% confidence interval of the RMSEA (RMSEA 90% CI) and its associated *p-*value (for RMSEA >0.05). To compare models, two additional fits were calculated, the Akaike information criterion (AIC; Akaike, 1987) and the modified expected cross-validation index (MECVI; Browne and Cudeck, 1989, 1993). Model selection was based on the lowest AIC and MECVI values.

**Figure 1:**
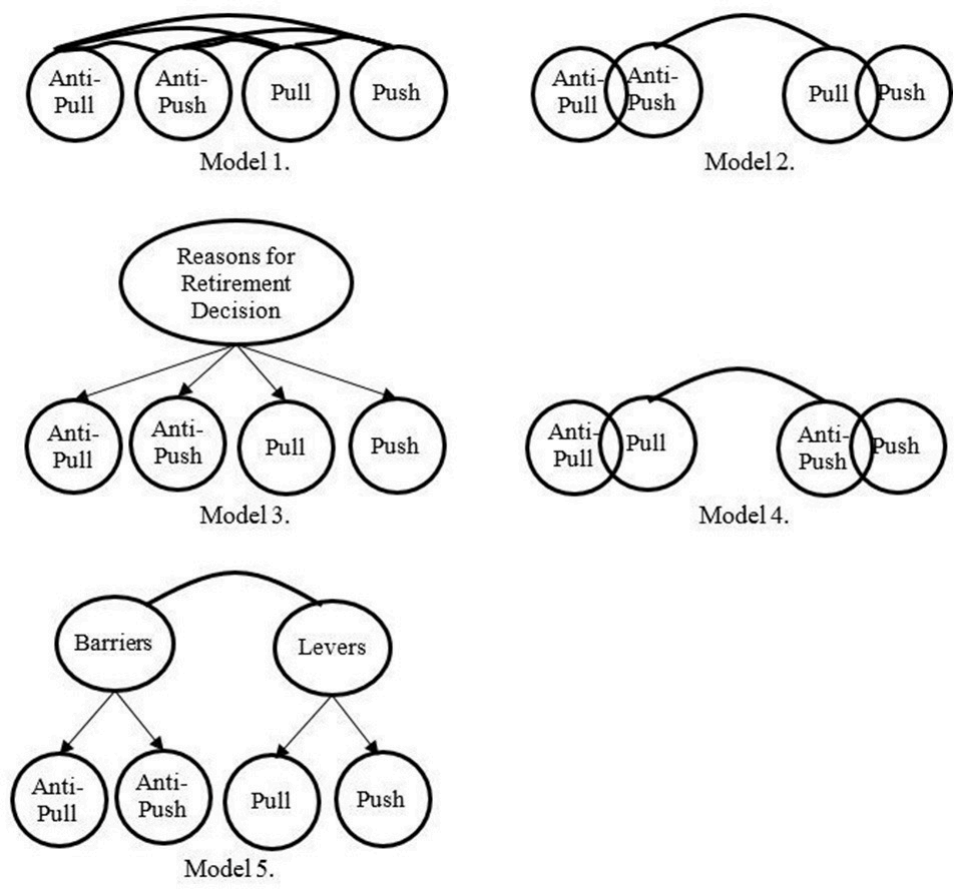
Competing models of the WRMI for study 3.

A series of models (Figure 1) was drawn up in accordance with the literature (Kim and Lee, 2002). In the first model (Figure 1, model 1), the 20 indicators loaded on four latent factors. Data conformed to the model with an acceptable fit index, B-Sχ^2^ = 189.14, df = 164, B-Sχ^2^/df = 1.15; CFI = 0.90; TLI = 0.90; SRMR = 0.06; RMSEA = 0.06, 90% CIs [0.05, 0.06], *p* = 0.39; AIC = 410.382; MECVI = 1.163. In addition, standardized regression weights were significant (λs > 0.48, *ps* < 0.001), exceeding the cutoff of 0.40 generally identified in the literature as a threshold value (Tinsley and Tinsley, 1987). In the second model (Figure 1, model 2), push and pull factors were collapsed as were anti-push and anti-pull factors, representing two first-order factors. Results indicated an inadequate fit of the model to the data, B-Sχ^2^ = 194.21, df = 169, B-Sχ^2^/df = 1.14; CFI = 0.59; TLI = 0.54; SRMR = 0.11; RMSEA = 0.11, 90% CIs [0.10, 0.12], *p* < 0.001; AIC = 1176.100; MECVI = 2.740. In the third model (Figure 1, model 3), the four first-order factors loaded on a second-order factor named retirement reasons. The results showed an inadequate fit of the model to the data, B-Sχ^2^ = 190.79, df = 167, B-Sχ^2^/df = 1.14; CFI = 0.87; TLI = 0.85; SRMR = 0.08; RMSEA = 0.06, 90% CIs [0.05, 0.06], *p* = 0.005; AIC = 485.564; MECVI = 1.371. In the fourth model (Figure 1, model 4), pull and anti-pull factors loaded on a second-order factor representing the present situation, and push and anti-push factors loaded on a second-order factor representing the future situation. The results showed an inadequate fit of the model to the data, B-Sχ^2^ = 190.78, df = 167, B-Sχ^2^/df = 1.14; CFI = 0.87; TLI = 0.85; SRMR = 0.09; RMSEA = 0.06, 90% CIs [0.05, 0.06], *p* = 0.004; AIC = 700.116; MECVI = 1.384. The fifth model (Figure 1, model 5) was tested with pull and push factors loading on a second-order factor representing levers, and anti-pull and anti-push factors loading on a second-order factor representing barriers. The results showed an inadequate fit of the model to the data, B-Sχ^2^ = 191.19, df = 167, B-Sχ^2^/df = 1.14; CFI = 0.88; TLI = 0.86; SRMR = 0.07; RMSEA = 0.06, 90% CIs [0.05, 0.06], *p* = 0.024; AIC = 675.245; MECVI = 1.315. Based on the fit of each model and on the two fit comparison tests (i.e., AIC and MECVI), the four-factor model (Figure 1, model 1) fit the data better than all the other models.

Overall, the results of Study 3 provide additional support for the four-factor model identified in Study 2, as well as for the reliability of the WRMI.

### Study 4: Social Desirability and Test-Retest Reliability

The first purpose of the fourth study was to assess the possible impact of social desirability on responses to the WRMI. This is an important step in scale validation as demonstrated by King and Bruner (2000). The second purpose was to investigate test-retest reliability.

#### Method

##### Participants

The sample consisted of 108 employees (62 male and 46 female) with an average age of 55.06 years (*SD* = 3.5; range 50–65 years), and average tenure of 32.19 years (*SD* = 6.30); 96 participants lived with a partner and 12 were single. Hundred and one participants worked full-time and 7 part-time. Of those 108 participants, 27 were contacted at random to complete a second inventory 15 days later. They were aged 52 to 62 years (*M* = 57.00; *SD* = 3.26) and only one worked part-time. Their average tenure as workers was 30.58 years (*SD* = 8.53).

##### Measures and procedure

*Reasons for retirement decision*. The internal consistency of the four dimensions of the WRMI was found to be acceptable, ranging from 0.79 to 0.92 at time 1, and from 0.88 to 0.95 at time 2 (Table 4). CRC was acceptable for each dimension, ranging from ρ = 0.82 to ρ = 0.94 at time 1, and ρ = 0.88 to ρ = 0.96 at time 2.

**Table 4:** Descriptive statistics for study 4.

	**Min**	**Max**	***M***	**SD**	**Skewness**	**Kurtosis**	**α**
T1desirability	2.00	11.00	7.20	2.09	0.60	−2.03	0.70
T1push	1.00	9.80	6.49	2.02	−3.66	0.67	0.86
T1pull	1.00	10.00	6.51	1.75	−1.59	0.31	0.75
T1anti–push	1.00	10.00	4.69	2.16	0.46	−1.54	0.84
T1anti–pull	1.00	9.40	4.72	2.38	0.65	−2.00	0.88
T2pull	1.40	10.00	5.77	2.07	0.32	0.20	0.87
T2push	1.40	9.60	6.28	2.00	−1.51	0.07	0.89
T2anti-push	1.00	9.40	3.92	2.25	2.06	0.47	0.90
T2anti-pull	1.00	9.00	4.01	2.37	1.33	−0.94	0.94

*Social desirability.* The short version (Form A) of the Marlowe-Crowne Social Desirability Scale (MCSDS; Crowne and Marlowe, 1960), validated by Loo and Thorpe (2000) was used to assess social desirability. This is a self-report questionnaire using a forced choice (True–False) format. Six questions were rescored, such that high values reflected a higher need to respond in a socially desirable manner. Hence, scores ranged from 0 (*no social desirability*) to 11 (*all socially desirable responses*). Internal consistency was acceptable (i.e., Cronbach's alpha.70). The questionnaires were completed under the conditions described in Studies 2 and 3, with a gap of 2 weeks between Time 1 and Time 2.

#### Statistical Analysis and Results

Neither univariate (i.e., |*z*| < 3.29, *p* < 0.001, two-tailed test) nor multivariate outliers (Mahalanobis distance lower than $\chi_{\left(5\right)}^{2}$ = 20.51, *p* < 0.001) were detected, and there were no missing values.

The first purpose of Study 4 was to assess whether the WRMI responses were biased by social desirability. Results of the correlation indicated that none of the four dimensions of the WRMI were significantly linked to social desirability (i.e., *r* ranging from −0.11 to 0.18 *ns*.).

The second purpose of the study was to assess the temporal stability of the WRMI. To this end, one-way intra-class correlations (ICCs) were calculated. Mean ICCs above 0.40 were acceptable, values of 0.60 or above indicated satisfactory stability, and values above 0.80 were considered as excellent (Landis and Koch, 1977).

Descriptive statistics are presented in Table 4. Supporting the temporal stability of the WRMI, ICCs for each subscale were as follows: push, *r* = 0.54; pull, *r* = 0.62; anti-push, *r* = 0.54; anti-pull, *r* = 0.84.

Thus, Study 4 indicates that the WRMI is not subject to social desirability and provides evidence for the scale's temporal stability as well as additional support for its reliability.

### Study 5: WRMI Invariance

The purpose of this study was to test in a new sample the degree to which WRMI is invariant for gender, marital status, contract, age and tenure, using the four-factor measurement model identified in Study 3 as the baseline model. Measurement invariance refers to the extent to which scores retain equivalent meaning across groups (Cheung and Rensvold, 2002). The above dimensions were chosen because they repeatedly emerge in previous empirical and theoretical literature as potential determinants of workers' career exit decisions (e.g., Beehr, 1986; Naudé et al., 2009; Wang and Shultz, 2010).

Tests of measurement invariance are thus important for future comparisons about the reasons underlying the retirement decisions of different groups of workers.

#### Method

##### Participants

The sample consisted of 433 workers (208 male and 225 female) with an average age of 56.27 years (*SD* = 3.69; range 50–66 years); 356 participants lived with a partner and 73 were single (4 participants did not answer this question). Average tenure of the 416 participants who provided this information (17 did not answer this question) was 27.59 years (*SD* = 11.21); 369 participants worked full-time and 64 part-time.

##### Measure

The internal consistency of the four dimensions of the WRMI was found to be acceptable, ranging from 0.74 to 0.90. The CRC was also acceptable, indicating good reliability (i.e., push, ρ = 0.81; pull, ρ = 0.75; anti-push, ρ = 0.87; anti-pull, ρ = 0.93).

#### Statistical Analysis and Results

Using Tabachnick and Fidell's (2013) recommendations, univariate (i.e., |*z*| < 3.29, *p* < 0.001, two-tailed test) and multivariate outliers (Mahalanobis distance lower than $\chi_{\left(4\right)}^{2}$ = 18.47, *p*<*0*.001) were discarded, leaving 331 participants. In this study, the amount of missing data was < 1% and was completely random (i.e., Little's χ^2^ = 252.58, df = 225, *p* = 0.06), allowing us to use EM algorithm imputations. The normalized Mardia's coefficient values for skewness (i.e., 122.74) with a criterion ratio of kurtosis multinormality above 1.96 (i.e., 42.95) indicated a significant multivariate non-normality of the data. Thus, all fit indices provided in these studies were based on Bollen-Stine bootstrap *p*-value and bootstrap-adjusted χ^2^ and goodness-of-fit indexes (Fouladi, 2000; Yuan and Hayashi, 2003).

In the following five stages, invariance of the WRMI was tested for gender (stage 1: men, *n* = 208, vs. women, *n* = 225), marital status (stage 2:living with a partner, *n* = 356, vs. single, *n* = 73), job-contract (stage 3: full-time, *n* = 369, vs. part-time, *n* = 64), age (stage 4: younger, *n* = 188, vs. older, n = 248), and tenure (stage 5: less experienced, *n* = 175, vs. more experienced, *n* = 239). Age and tenure were dichotomized in two categories based on median values, respectively 55 and 30 years. The invariance routine involved tests and comparisons of nested models that imposed successive restrictions on model parameters. Equivalence of covariance structure was investigated using the sequential order proposed by Marsh et al. (2005). Equivalence of mean structure was investigated using the sequential order proposed by Byrne et al. (1989). Both equivalences were tested using the sequential steps proposed by Meredith (1993): (i) configural invariance or invariance of the factor structure representing the baseline model (Model 1), (ii) equality constraints on the factor loadings, or weak invariance (Model 2), (iii) equality constraints on the items' intercepts, or strong invariance (Model 3), (iv) equality constraints on uniqueness of items, or strict invariance (Model 4), (v) equality constraints on the variance of the latent factors (Model 5), (vi) equality constraints on the covariance between the latent factors (Model 6), and (vii) equality constraints of the latent factor means, or total invariance (Model 7).

Adequacy of fit for the models was based on multiple indicators: the Bollen-Stine χ^2^, the B-Sχ^2^/df ratio, the CFI, the TLI, the SRMR, the RMSEA and the 90% confidence interval of the RMSEA (RMSEA 90% CI) and its associated *p-*value (for RMSEA < 0.05). Concerning gender invariance (stage 1), results (Table 5) indicated an adequate fit of the model to the data for the least restricted model (Model 1). This baseline model combined the male and female samples established in the single-group CFA models with no cross-group equality constraints. Results indicated that the WRMI has the same number of common factors across groups. Fixing item loadings to test weak invariance (Model 2) led to a non-significant chi-square increase ΔS-B$\chi_{\left(16\right)}^{2}$ = 15.430 and a marginal change for CFI and RMSEA. Fixing item intercepts (Model 3) resulted in a non-significant Chi-square increase ΔS-B$\chi_{\left(32\right)}^{2}$ = 31.624, and CFI and RMSEA delta < 0.01 and 0.015, respectively. Placing cross-group equality constraints on item uniqueness to test strict invariance (Model 4) led to a non-significant chi-square increase ΔS-B$\chi_{\left(52\right)}^{2}$ = 63.723 and minimal differences of CFI and RMSEA. Fixing variance (Model 5) and covariance (Model 6) led to a non-significant chi-square increase ΔS-B$\chi_{\left(56\right)}^{2}$ = 66.919 and ΔS-B$\chi_{\left(62\right)}^{2}$ = 74.603, respectively, and minimal differences of CFI and RMSEA indicating variance and covariance invariance. Results were similar when mean latent factors were fixed (Model 7), with a non-significant chi-square increase ΔS-B$\chi_{\left(66\right)}^{2}$ = 78.400 and minimal differences of CFI and RMSEA.

**Table 5:** Goodness of Fit Indices of WRMI models for study 5.

**Stage**	**Model**	**Description**	**B-S _χ_^2^**	**df**	**CFI**	**TLI**	**SRMR**	**RMSEA**	**RMSEA 90% CI**	**ΔB-S_χ_^2^**	**Δdf**	**|ΔCFI|**	**|ΔRMSEA|**
Stage 1	**Gender-invariance**												
	Model 1	Configural invariance	431.954^*^	328	0.904	0.888	0.0757	0.053	[0.047, 0.058]				
	Model 2	λs invariant	447.384^*^	344	0.905	0.894	0.0762	0.051	[0.046, 0.056]	15.430	16	−0.001	−0.002
	Model 3	λs, τs invariant	463.578^*^	360	0.901	0.894	0.0760	0.051	[0.046, 0.056]	31.624	32	−0.003	−0.002
	Model 4	λs, τs, δs invariant	495.677^*^	380	0.899	0.899	0.0760	0.050	[0.045, 0.055]	63.723	52	−0.005	−0.003
	Model 5	λs, τs, δs, ξs invariant	498.873^*^	384	0.899	0.899	0.0778	0.050	[0.045, 0.055]	66.919	56	−0.004	−0.003
	Model 6	λs, τs, δs, ξs, ϕs invariant	506.557^*^	390	0.899	0.901	0.0794	0.049	[0.044, 0.054]	74.603	62	−0.005	−0.004
	Model 7	λs, τs, δs, ξs, ϕs, ηs invariant	510.354^*^	394	0.897	0.900	0.0786	0.050	[0.044, 0.055]	78.400	66	−0.007	−0.003
Stage 2	**Marital status–invariance**												
	Model 1	Configural invariance	457.390^*^	328	0.915	0.901	0.0809	0.049	[0.044, 0.055]				
	Model 2	λs invariant	472.785^*^	344	0.916	0.908	0.0860	0.048	[0.043, 0.053]	15.395	16	+0.001	−0.001
	Model 3	λs, τs invariant	489.061^*^	360	0.915	0.910	0.0860	0.047	[0.042, 0.052]	31.671	32	0.000	−0.002
	Model 4	λs, τs, δs invariant	516.459^*^	380	0.913	0.913	0.0889	0.046	[0.041, 0.052]	59.069	52	0.000	−0.003
	Model 5	λs, τs, δs, ξs invariant	519.394^*^	384	0.914	0.914	0.0914	0.046	[0.041, 0.051]	62.004	56	−0.002	−0.003
	Model 6	λs, τs, δs, ξs, ϕs invariant	526.044^*^	390	0.914	0.916	0.1036	0.046	[0.041, 0.051]	68.654	62	−0.002	−0.003
	Model 7	λs, τs, δs, ξs, ϕs, ηs invariant	530.019^*^	394	0.914	0.917	0.1035	0.045	[0.040, 0.050]	72.629	66	−0.002	−0.004
Stage 3	**Contract-invariance**												
	Model 1	Configural invariance	473.423^*^	328	0.917	0.904	0.0631	0.048	[0.043, 0.054]			
	Model 2	λs invariant	488.799^*^	344	0.919	0.910	0.0635	0.047	[0.042, 0.052]	15.376	16	−0.002	−0.001
	Model 3	λs, τs invariant	505.102^*^	360	0.920	0.916	0.0634	0.045	[0.040, 0.051]	31.679	32	+0.003	−0.003
	Model 4	λs, τs, δs invariant	532.222^*^	380	0.920	0.920	0.0633	0.044	[0.039, 0.049]	58.799	52	+0.003	−0.004
	Model 5	λs, τs, δs, ξs invariant	535.644^*^	384	0.920	0.921	0.0634	0.044	[0.039, 0.049]	61.221	56	+0.003	−0.004
	Model 6	λs, τs, δs, ξs, ϕs invariant	542.671^*^	390	0.919	0.921	0.0638	0.044	[0.039, 0.049]	69.248	62	+0.002	−0.004
	Model 7	λs, τs, δs, ξs, ϕs, ηs invariant	546.745^*^	394	0.919	0.922	0.0639	0.044	[0.038, 0.049]	73.322	66	+0.002	−0.004
Stage 4	**Age-invariance**												
	Model 1	Configural invariance	442.123^*^	328	0.903	0.898	0.0734	0.051	[0.046, 0.057]			
	Model 2	λs invariant	457.202^*^	344	0.909	0.900	0.0729	0.050	[0.045, 0.055]	15.079	16	+0.006	−0.001
	Model 3	λs, τs invariant	469.694^*^	360	0.911	0.905	0.0729	0.049	[0.043, 0.054]	27.571	32	+0.008	−0.002
	Model 4	λs, τs, δs invariant	501.947^*^	380	0.908	0.908	0.0736	0.048	[0.043, 0.053]	59.824	52	+0.005	−0.002
	Model 5	λs, τs, δs, ξs invariant	504.883^*^	384	0.908	0.909	0.0744	0.047	[0.042, 0.052]	62.730	56	+0.005	−0.004
	Model 6	λs, τs, δs, ξs, ϕs invariant	512.323^*^	390	0.905	0.908	0.0858	0.048	[0.043, 0.053]	70.200	62	+0.002	−0.003
	Model 7	λs, τs, δs, ξs, ϕs, ηs invariant	516.245^*^	394	0.905	0.909	0.0855	0.048	[0.043, 0.052]	74.122	66	+0.002	−0.003
Stage 5	**Tenure-invariance**												
	Model 1	Configural invariance	434.620^*^	328	0.901	0.885	0.0862	0.053	[0.048, 0.059]			
	Model 2	λs invariant	449.668^*^	344	0.902	0.892	0.0836	0.052	[0.046, 0.070]	15.048	16	+0.001	−0.001
	Model 3	λs, τs invariant	462.315^*^	360	0.900	0.894	0.0835	0.051	[0.046, 0.056]	27.695	32	−0.001	−0.002
	Model 4	λs, τs, δs invariant	494.907^*^	380	0.901	0.901	0.0831	0.049	[0.044, 0.054]	60.287	52	0.000	−0.004
	Model 5	λs, τs, δs, ξs invariant	497.912^*^	384	0.901	0.902	0.0841	0.049	[0.044, 0.054]	63.292	56	0.000	−0.004
	Model 6	λs, τs, δs, ξs, ϕs invariant	506.164^*^	390	0.901	0.903	0.0896	0.049	[0.044, 0.054]	71.544	62	0.000	−0.004
	Model 7	λs, τs, δs, ξs, ϕs, ηs invariant	510.184^*^	394	0.901	0.905	0.0896	0.049	[0.043, 0.054]	75.56	66	0.000	−0.004

*B-S_χ_^2^, Bollen-Stine Chi-square; df, Degrees of freedom; CFI, Comparative fit index; TLI, Tucker-Lewis index; SRMR, Standardized root mean square residual; RMSEA, Root mean square error of approximation; RMSEA 90% CI, 90% Confidence interval for the RMSEA point estimate; λ, Factor loading; τ, Intercept; δ, Uniqueness; ξ, Factor variance; ϕ, Factor covariance; η, Factor mean; ΔB-S_χ_^2^, Change in goodness-of-fit B-S_χ_^2^ relative to the baseline model; Δdf, Change in degrees of freedom relative to the baseline model; ΔCFI, Change in comparative fit index relative to the baseline model; ΔRMSEA, Change in root mean square error of approximation relative to the baseline model*;

* *p < 0.05*.

The same procedure was followed for marital status (Stage 2), contract (Stage 3), age (Stage 4), and tenure (Stage 5); the results are presented in Table 5. In all stages, results indicated full invariance with non-significant chi-square changes and minimal differences of CFI and RMSEA. These results indicate that the WRMI is a fairly consistent measurement across gender, marital status, contract, age, and tenure.

### Study 6: WRMI Predictive Validity

Because the major interest of the WRMI is its ability to identify the multidimensional psychosocial process underlying retirement decision-making, the two-fold aim of the final study was (a) to address the simultaneous associations of the four dimensions of the model (i.e., Push, Pull, Anti-Push, Anti-Pull) within individual workers by adopting a person-centered approach, and (b) to investigate associations between the profiles and three indicators of the intention to stop the professional career (i.e., planned retirement age, attitudes toward retirement, and intention toward planned retirement).

#### Method

##### Participants

The sample of this last study was composed of 304 male and 383 female workers. The average age was 56.87 years (*SD* = 2.95), ranging from 50 to 66 years; 517 participants lived with a partner and 166 lived alone (4 did not answer this question). Average working tenure was 23.18 years (*SD* = 11.44). Finally, 567 employees worked full-time and 119 part-time (one did not answer this question).

##### Measures

*WRMI*. Participants completed the 20-item inventory. Results of the CFA again provided support for the internal validity of the model, B-Sχ^2^ = 214.614, df = 164, B-Sχ^2^/df = 1.308; CFI = 0.951; TLI = 0.943; SRMR = 0.038; RMSEA = 0.052, 90% CIs [0.047, 0.057], *p* = 0.265; AIC = 610.519; MECVI = 0.762. The internal consistency of the four dimensions of the WRMI were found to be acceptable, α = 0.83, α = 0.75, α = 0.85, α = 0.89, for push, pull, anti-push, and anti-pull, respectively. The CRC was acceptable for each dimension, ρ = 0.83, ρ = 0.79, ρ = 0.85, and ρ = 0.89 for push, pull, anti-push, and anti-pull, respectively.

*Planned retirement age*. Planned retirement age was assessed by a single item: “When do you intend to retire?” This use of a single-item variable is common in retirement research (e.g., Beehr et al., 2000). Based on previous studies (e.g., Taylor and Shore, 1995; Griffin et al., 2012), we calculated the difference between the planned retirement age and the participants' current age, higher scores representing a longer period between the current age and the planned retirement age.

*Planned retirement attitudes and intentions*. Based on Marcil et al.'s (2001) questionnaire, attitudes toward planned retirement (7 items, e.g., “It would be sensible to plan my retirement”) and intention to plan retirement (4 items, e.g., “I intend to plan when I retire”) were assessed separately. Participants responded on a 7-point Likert-type scale ranging from 1 (*strongly agree*) to 7 (*strongly disagree*). Thus, a low score indicated positive attitudes and intentions. Results of the CFA showed an adequate fit of the model to the data, B-Sχ^2^ = 63.299, df = 43, B-Sχ^2^/df = 1.47; CFI = 0.932; TLI = 0.914; SRMR = 0.056; RMSEA = 0.085, 90% CIs [0.075, 0.096], *p* < 0.001; AIC = 303.812; MECVI = 0.444. The CRC and Cronbach's alpha were acceptable for each dimension, with α = 0.82 and ρ = 0.85 for intention, and α = 0.85 and ρ = 0.90 for attitudes toward planned retirement.

#### Statistical Analysis and Results

##### Latent profile analysis

Neither univariate nor multivariate outliers were found. Missing data amounted to < 1% and were completely random (i.e., Little's χ^2^ = 1548.252, df = 12231, *p* = 1.000), enabling us to use the EM algorithm imputations. Descriptive statistics are presented in **Table 7**.

Latent profile analyses (LPAs) were performed with Mplus Version 8.1 (Muthén and Muthén, 1998–2015) to identify groups of workers who showed similar patterns on the Push Pull Anti-push Anti-pull dimensions. Solutions of 1–4 classes were tested to identify the ideal number of classes. Model fit criteria were inspected across solutions to determine the best fit to the data. In a first step, an adjusted Lo-Mendell-Rubin likelihood ratio test (LMR; Lo et al., 2001) was used to compare the fit of two models. Classes were added iteratively to identify the best model fit. A significant LMR test (*p* < 0.05) indicates that the target class solution fits better with the data than a class solution with one fewer class. In a second step, the Akaike Information Criterion (AIC; Akaike, 1987), the Bayesian Information Criterion (BIC; Schwarz, 1978) and the sample-size adjusted BIC (SSA-BIC; Sclove, 1987) were inspected, with lower scores representing better fitting models. The entropy criterion was also examined, which indicates how accurate people are classified into their respective profiles. Entropy values range from 0 to 1; higher values indicate a better fit for a given solution (Aldridge and Roesch, 2008). Finally, analyses of variance tests (ANOVAs) were performed to interpret the latent profile analysis solution from a theoretical point of view.

After the hypothesized groups were identified, ANOVAs were conducted to examine the associations between profile membership and three indicators of the intention to stop the professional career (i.e., planned retirement age, attitudes toward retirement and intention toward planned retirement).

Latent profile models containing 1, 2, 3, and 4 classes were fit to the data. The model fit indices for each LPA are available in Table 6. The adjusted LMR test indicated that the 2-class solution fit better than the 1-class solution (*p* < 0.001). The 3-class solution was deemed superior to the 2-class solution due to a significant LMR test (*p* = 0.001), lower AIC, BIC, and SSA-BIC values, and higher entropy value. Although the 4-class solution revealed lower AIC, BIC and SSA-BIC values, the entropy value is slightly lower and the LMR test indicated that it was not statistically different from the 3-class solution (*p* = 0.234). Therefore, the 3-class solution was considered the best fit to the data.

**Table 6:** Fit indices, entropy, and model comparisons for estimated latent profile analyses models (study 6).

**Models**	**AIC**	**BIC**	**SSA-BIC**	**Entropy**	**Adjusted LMR test**
1 Class	7745.18	7781.42	7756.01	__	__
2 Classes	7334.80	7393.69	7352.41	0.76	407.88^***^
**3 Classes**	**7131.25**	**7212.78**	**7155.63**	**0.80**	207.20^**^
4 Classes	7047.25	7151.43	7078.40	0.79	91.21 *ns*

*AIC, Akaike Information Criterion; BIC, Bayesian Information Criterion; SSA-BIC, Sample-Size Adjusted Bayesian Information Criterion; Adjusted LMR test, Adjusted Lo-Mendell-Rubin likelihood ratio test. Bold corresponds to the model with the best fit*.

** *p < 0.01*,

*** *p < 0.001, ns, non-significant*.

**Figure 2:**
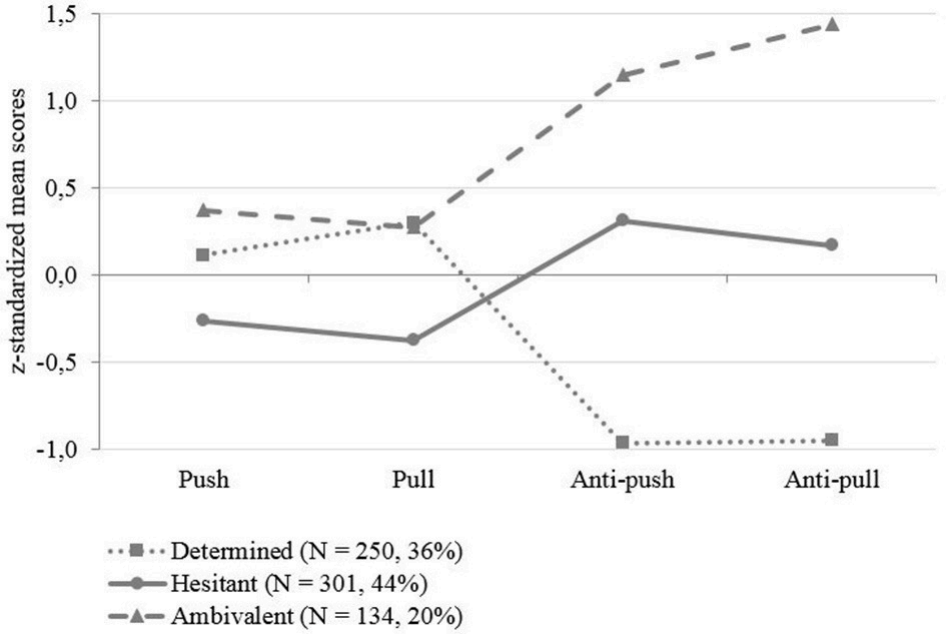
Classes through latent profile analysis (study 6).

The three latent profile classes are depicted graphically in Figure 2. To help in the interpretation of the classes, the standardized means for each profile on Push, Pull, Anti-push, and Anti-pull are represented.

A MANOVA revealed that the subgroups of the three-class solution differed significantly on all the WRMI dimensions (Wilk's λ = 0.123, *F*_(8, 1340)_ = 309.383, *p* < 0.001, partial η^2^ = 0.649).

Results of the univariate ANOVAs indicated that the subgroups of the three-class solution differed significantly on all the WRMI dimensions. *Post-hoc* Tukey tests were conducted to explore pairwise comparisons between classes on Push Pull Anti-push Anti-pull dimensions. The observed significant differences enabled us to establish “high,” “medium,” or “low” criteria for each variable. Descriptive statistics for each class are presented in Table 7.

**Table 7:** Descriptive statistics for the latent profile classes (study 6).

	**Total** ***n*** = **685**		**Determined seniors** ***n*** = **250**		**Hesitant seniors** ***n*** = **301**		**Ambivalent seniors**		***F*** ***n*** = **134**	**η^2^**
	***M***	***SD***	***M***	***SD***	***M***	***SD***	***M***	***SD***		
Push	6.36	1.99	6.57	2.19	5.82	1.79	7.15	1.68	24.61^***^	0.07
Pull	7.05	1.82	7.60	1.71	6.34	1.77	7.60	1.54	45.99^***^	0.12
Anti-push	4.32	2.15	2.17	1.01	4.98	1.30	6.83	1.43	702.88^***^	0.67
Anti-pull	4.38	2.43	2.00	0.97	4.76	1.30	7.93	1.02	1214.60^***^	0.78
Planned age	5.76	3.71	4.86	3.08	6.69	4.07	5.36	3.44	18.68^***^	0.05
Intention	2.98	1.51	2.83	1.56	3.16	1.44	2.87	1.56	3.68^*^	0.01
Attitude	2.91	1.18	2.57	1.14	3.19	1.13	2.93	1.21	20.05^***^	0.06

* *p < 0.05*,

*** *p < 0.001*.

The first class (*n* = 250), labeled “*determined seniors*,” showed a medium Push level, a high Pull level and low Anti-push and Anti-pull levels. Pairwise comparisons indicated significant differences between these scores and those in the other classes (all *p*s < 0.05), with the exception of the Pull dimension, which did not differ significantly from class 3.

We called the second class (*n* = 301) “*hesitant seniors*.” They reported low Push and Pull levels and medium Anti-push and Anti-Pull levels. Pairwise comparisons indicated significant differences between their scores and those in the other classes (all *p*s < 0.001).

The third class (*n* = 134) was labeled “*ambivalent seniors*.” Workers in this class attributed high importance to the four factors, with values differing significantly from those given by workers in the other classes (*p*s < 0.05), except for Pull perception in class 1.

##### Differences in planned retirement age, attitudes and intentions between classes

Class differences in planned retirement age were examined. The age of the participants was controlled. Results of the ANOVA indicated that the three classes differed significantly, *F*_(2, 681)_ = 37.352, *p* < 0.001, partial η^2^ = 0.099. Tukey *post-hoc* tests showed that the “*determined seniors*” subgroup had significantly lower scores for planned retirement age (Zmean = −0.245) than the “*hesitant”* (Zmean = 0.251; *p* < 0.001) and “*ambivalent”* (Zmean = −0.108; *p* < 0.01) subgroups. The “*hesitant seniors*” subgroup had significantly (*p* < 0.001) higher values for planned retirement age than the other two subgroups. Finally, the “*ambivalent*” subgroup came significantly between the other two subgroups. In summary, these results show that class 1 (i.e., “*determined seniors*”) was related to the shortest period between current age and planned retirement age, as opposed to classes 2 and 3 (i.e., “*hesitant seniors*” and “*ambivalent seniors*”).

Next we examined class differences in attitudes toward planned retirement. The age of the participants was controlled. Results of the ANOVA indicated that the three classes differed significantly in their attitudes, *F*_(2, 674)_ = 21.109, *p* < 0.001, partial η^2^ = 0.059. The “*determined*” subgroup had significantly lower scores (Zmean = −0.291) than the “*hesitant”* (Zmean = 0.239; *p* < 0.001) and “*ambivalent”* (Zmean = 0.012; *p* < 0.01) subgroups. The “*hesitant”* subgroup had significantly higher scores than the “determined” (*p* < 0.001) and “ambivalent” (*p* < 0.05) subgroups. Finally, the “*ambivalent”* subgroup came significantly between the other two subgroups. In brief, these results show that class 1 (i.e., “*determined seniors*”) was related to positive attitudes toward the retirement, as opposed to classes 2 and 3 (i.e., “*hesitant seniors*” and “*ambivalent seniors*”).

Finally, we examined class differences in planned retirement intentions. The age of the participants was controlled. Results of the ANOVA showed that the three classes differed significantly, *F*_(2, 677)_ = 3.878, *p* < 0.05, partial η^2^ = 0.011. The “*hesitant”* subgroup had significantly (*p* < 0.01) higher scores (Zmean = 0.118) than the determined subgroup (Zmean = −0.098). No differences were found between the “*ambivalent*” subgroup (Zmean = −0.075) and the “hesitant” (*p* = 0.067) or “determined” (*p* = 0.752) subgroups. In brief, these results indicate that class 2 (i.e., “*hesitant seniors*”) was related to negative retirement planning intentions, as opposed to class 1 (i.e., “*determined seniors*”).

## General Discussion

It is widely recognized in the relevant literature today that the retirement decision process is an individually motivated choice based on different types of reasons (Wang and Shultz, 2010; Wang and Shi, 2014). Some researchers have therefore tried to clarify this issue by developing various models to structure the diversity and nature of these potential factors underlying the decision to retire (e.g., Wang and Shultz, 2010; Wang and Shi, 2014). One of the most popular approaches involves differentiating between push and pull factors (Lund and Villadsen, 2005; De Preter et al., 2013; Bayl-Smith and Griffin, 2014). Push factors are those that create pressure to leave work, whereas pull factors relate to aspects that positively motivate one to retire (Beehr and Bennett, 2007). Such general classifications are very useful, providing a comprehensive picture of the causes underlying the retirement decision, but they need to be approached with caution as they can over-simplify a complex and multi-dimensional phenomenon. Indeed, empirical reports have revealed that the decision to retire results more from a subjective balance between different types of consideration than from isolated reasons. For example, Schlossberg (2009) observed that individuals making retirement decisions weigh the pros and cons of their current life situation against their imagined “future” in retirement. For that reason, some authors (e.g., Schlossberg, 2009; Chevalier et al., 2013) recently concluded that a multi-dimensional approach to studying the retirement decision is preferable to a single domain approach. However, to date, very few studies have measured this decision as a multifaceted construct, probably due to the lack of accurate measures. Thus, the aim of the present study was to overcome an important design weakness of most studies of retirement decision-making by developing a new reliable multifaceted measure specifically designed to capture the complexity of the psychosocial mechanisms underlying the individual process whereby senior workers decide to end or continue their careers. More precisely, six studies were conducted to develop and test the WRMI inspired by the push pull anti-push anti-pull model developed by Mullet et al. (2000). The aim of Study 1 was to create a pool of items reflecting the main positive and negative aspects of the present and future situation that play a role in senior workers' retirement decisions. Study 2 clearly established the underlying four-factor dimensionality of the new tool, corresponding to the theoretically relevant categories, and confirmed in a large sample in Study 3. The first factor (Push) involved negative views of the worker's present life. The second factor (Anti-pull) included the overall risk and cost aspects of the post-career life. The third factor (Pull) corresponded to the positive aspects of the post-career life. The fourth factor (Anti-push) referred to attachment to the professional career. The four-factor model explained a large proportion of the total variance. Study 4 demonstrated that WRMI is not subject to social desirability bias, and the 2-week design demonstrated its temporal stability. Study 5 examined the metric invariance of the scale across gender, marital status, type of contract, age, and tenure. Multigroup analyses supported the reliability, factorial structure, and validity of the WRMI across these different groups. Finally, Study 6 aimed to identify motivational profiles of the participants resulting from the different weights given to the four dimensions and then to examine how these different profiles were associated with three indicators of the retirement decision (i.e., planned retirement age, attitudes, and intentions). The results revealed a three-class solution. The first class, which we called “*determined seniors*,” was composed of workers who attributed rather high importance to the levers of the retirement decision (a medium score on the Push factor and a high score on the Pull dimension) and low importance to the Anti-push and Anti-pull dimensions, both considered as barriers to the retirement decision. The second class, “*hesitant seniors*,” was composed of workers with a low score on Push and Pull factors (i.e., levers of the retirement decision), and a medium score on the barriers to retirement (i.e., Anti-push and Anti-pull dimensions). The third class, “*ambivalent seniors*,” included workers who attributed high importance to both the levers and the barriers of the retirement decision. With regard to the second objective of study 6, we observed significantly different associations between the three profiles and planned retirement age, attitudes and intentions.

In summary, all the results presented herein support the psychometric properties of this new measure of workers' reasons for retiring, although further improvements are required. For example, to complete the validation work, it would be interesting to explore the convergent validity of the four subscales in relation to conceptually related constructs such as work engagement (Schaufeli et al., 2006) or work amotivation (Gagné et al., 2010) for push and anti-push factors, and attitudes toward retirement (Anson et al., 1989) or anxiety about retirement (Hayslip et al., 1997) for pull and anti-pull factors. Nevertheless, the WRMI can already be considered to be a valid and innovative tool that can be used fruitfully for research and counseling. First, using this new multidimensional inventory based on the integrative Push Pull Anti-Push Anti-Pull model would give researchers the opportunity to examine both present vs. future levers (i.e., Push Pull) and present vs. future barriers (Anti-push Anti-pull) in the retirement decision and above all to examine how the four dimensions operate in conjunction with each other. Indeed, empirical observations demonstrate that our inventory provides a comprehensive view of how workers consider more than one dimension as they approach retirement and may oscillate between Push, Pull, Anti-push, and Anti-pull factors. Consequently, the WRMI could be used in future research to study in-depth the psychological management of career termination.

Secondly, the advantage of this multidimensional tool over previous unidimensional questionnaires is that it combines a variable-centered analysis and a person-centered approach. And while it is important for this research area to clarify how the importance of each WRMI dimension varies among senior workers (i.e., to capture the nuances and the complexity of inter-individual variations within a system of variables; Meyer et al., 2013), it is also fundamental for the retirement decision literature to clarify how the four types of reason are assessed as a whole and work in different combinations for different subgroups of workers. Indeed, once subgroups of individuals sharing similar profiles on the four dimensions have been identified, researchers could examine whether these groups differ on other criteria (e.g., resources, Leung and Earl, 2012) and outcomes (e.g., retirement preparedness) in theoretically predictable ways. This could help overcome some inconsistent current results (e.g., Radl, 2013) by determining precisely how the behavior of some senior workers (e.g., retirement timing, retirement planning) depends less on the relative importance of each type of reason than on their combination. For example, our preliminary results show that classes 1 and 3 (i.e., “*determined*” and “*ambivalent*”) included senior workers with a higher level of retirement planning and attitudes toward planned retirement than class 2 (i.e., “*hesitant*”). This is in line with existing literature (e.g., De Preter et al., 2013), showing that high scores on traditional Push and Pull factors are linked to the positive attitudes of senior workers facing the transition to retirement. However, they also demonstrate that the influence of these two dimensions on the intention to retire is stronger when the importance attributed to Anti-push and Anti-pull factors is low. Indeed, the difference between the “*determined*” and “*ambivalent*” classes concerns essentially the importance attributed to Anti-push and Anti-pull factors (i.e., low in the “*determined*” and high in the “*ambivalent*” class).

In brief, adopting the person-centered and variable-centered strategies provided by our tool in future studies could offer new insight into the variety of cognitive mechanisms involved in the retirement decision process and in their associated behaviors.

Finally and more globally, the WRMI could be used to enrich theoretical hypotheses relative to other relevant models in the retirement research field. For example, comparing the approach-avoidance motivation theory (Shultz et al., 1998; Gobeski and Beehr, 2009) and the Push Pull Anti-Push Anti-Pull model may be particularly useful for a better understanding of why so many older workers are ambivalent about the prospect of retirement (Feldman and Beehr, 2011). Similarly, using the WRMI in future studies based on the temporal perspective of retirement (Wang, 2007; Löckenhoff et al., 2009) could allow researchers to test some major conclusions of the model. For example, Feldman (1994) and Feldman and Beehr (2011) showed that the amount of past-, future-, and present-oriented thinking about retirement varies across the retirement decision-making process. In a recent study, Maggiori et al. (2014) found evidence that the number of years to or from the retirement transition has an impact on the perception of the transition to retirement. In short, their results indicate that the perception of and plans for retirement evolve during the years before and after the transition. Use of the WRMI to study the pattern of reasons underlying the retirement decision could add to our understanding of the various outcomes of the different stages of the retirement decision.

From an applied perspective, understanding the reasons that encourage or discourage retirement is crucial for governments and organizations having to cope with an aging workforce. Indeed, extending people's working life is seen as a key element in dampening or curtailing the rising costs associated with an aging population (Van Solinge and Henkens, 2014).

The WRMI is the first validated instrument to provide practitioners with an overall view of the reasons, beyond the commonly used Push and Pull dimensions, which can lead senior workers to end or continue their careers, and consequently help prepare these people for their final career transition. Indeed, this new parsimonious inventory could be used in counseling situations as a relatively rapid screening device to refine the diagnosis and to help draw up appropriate intervention strategies. For instance, a worker with a WRMI profile characterized by many worries about post-career life would benefit from counseling targeted at reducing anxiety. Similarly, workers approaching retirement age and who are still strongly committed to their work would benefit from counseling focused on developing new interests and activities. Moreover, if senior workers give importance to Push or Pull factors for example, this alone will not determine whether they are really ready to retire. The counselor needs to examine whether the importance given to the barriers (Anti-push and Anti-pull) is also low (i.e., “determined” class). It is thus crucial for counselors helping senior workers prepare for retirement to be aware that this decision process is multifaceted, complex and individual.

The WRMI could also be particularly useful to assess sensitivity to individual change following an intervention aimed at retirement planning, through repeated measures of the importance given over time to present vs. future levers (i.e., Push Pull) and present vs. future barriers (i.e., Anti-push Anti-pull) in the retirement decision.

Finally, workers themselves should benefit from a better understanding of the reasons underlying their motivation to retire or to postpone the decision to end their career. This could be facilitated by the perspective provided by the four-dimensional aspect of the WRMI. This instrument could thus prove to be of great value in both research and practice.

Nevertheless, while this current study was based on a rigorous empirical procedure, namely, multiple independent samples to develop and validate the WRMI, some limitations should be mentioned. First, it may not be generalizable to all senior workers and all contexts. Indeed, this was not a national cross-section of older employees, but was based on convenience samples. The extent to which the results generalize to other French groups or countries is currently unknown. Furthermore, the national environment may influence the retirement decision (Hershey et al., 2007; Kim, 2008; Wang and Shultz, 2010), and it is possible that the cultural context of France produces distinct profiles that would not be observed in other countries and that the observed effects of the profiles on intention to retire would not be replicated in other contexts. Thus, representative samples of other countries should be included in future research. The second limitation is linked to the heterogeneity of our samples with regard to some individual characteristics (e.g., personality traits, level of education, age) and professional dimensions (e.g., quality of life at work, workplace interactions). While our objective was not to test the role of these variables, future research should provide further evidence to validate the factorial structure of the WRMI among samples that vary across these characteristics, and more generally it should expand the scope of the antecedents and outcomes associated with the different retirement motivation profiles. Thirdly, the data of Study 6 were cross-sectional, thereby limiting inferences of causality between the three identified classes and the three indicators of the retirement decision. To overcome this problem, future research should use a longitudinal design aimed at better identifying the causal direction.

In conclusion, we hope that the WRMI will help the proliferation of studies that use the push pull anti-push anti-pull model for research on retirement decision-making. Indeed, like other researchers (e.g., Feldman, 1994; Shultz and Wang, 2011), we are convinced that individuals may consider a wide variety of factors simultaneously when making their decision. In other words, and more precisely, the retirement decision is the result of the importance given to present vs. future levers (i.e., Push Pull) and present vs. future barriers (i.e., Anti-push Anti-pull) acting together. Consequently, the four-dimensional model is particularly appropriate to investigate this complex process.

## Data Availability Statement

The raw data supporting the conclusions of this manuscript will be made available by the authors, without undue reservation, to any qualified researcher.

## Author Contributions

EF, GB, and SC contributed conception and design of the study. EF, GB, SC, VD, CB, and NG organized the database. EF, GB, SC, and HC performed the statistical analysis. EF and GB wrote the first draft of the manuscript. All authors contributed to manuscript revision, read and approved the submitted version.

### Conflict of Interest Statement

The authors declare that the research was conducted in the absence of any commercial or financial relationships that could be construed as a potential conflict of interest.
