# Supplementary material for: Better Understanding the Workers' Retirement Decision Attitudes: Development and Validation of a New Measure
Source: Front Psychol. 2018 Dec 4;9:2429. doi: 10.3389/fpsyg.2018.02429 (PMC6290337; doi:10.3389/fpsyg.2018.02429)
Supplement: Supplementary file 1 [file Data_Sheet_1.PDF]

Supplementary Material

Better Understanding the Workers' Retirement Decision Attitudes: Development and Validation of a New Measure

Evelyne Fouquereau*, Grégoire Bosselut, Séverine Chevalier, Hélène Coillot, Virginie Demulier, Caroline Becker, Nicolas Gillet

*** Correspondence:** Evelyne Fouquereau: evelyne.fouquereau@univ-tours.fr

Workers' Retirement Motivations Inventory.

1. Push

1a. Feeling less motivated at work

1b. Feeling that the work atmosphere is not pleasant

1c. Having the impression that my work conditions have deteriorated

1d. Feeling stressed by my job

1e. Feeling dissatisfied with my work conditions

2. Pull

2a. Being able to spend more time with my family when I retire.

2b. Being able to relax when I retire.

2c. Being able to control my personal life better when I retire.

2d. To be under less pressure in general when I retire.

2e. Being able to spend more time with my friends when I retire.

3. Anti-push

3a. Being attached to my companies

3b. Feeling that I can still play an active role at work.

3c. Being attached to my professional status

3d. Still having professional ambitions

3e. Feeling that my professional work gives me social recognition

4. Anti-pull

4a. Being afraid of losing my energy when I retire

4b. Being afraid of feeling depressed when I retire

4c. Being afraid of growing old quickly when I retire

4d. Being afraid of feeling lonely when I retire

4e. Being afraid of being bored when I retire
